# TNFAIP8 promotes AML chemoresistance by activating ERK signaling pathway through interaction with Rac1

**DOI:** 10.1186/s13046-020-01658-z

**Published:** 2020-08-14

**Authors:** Yihua Pang, Yanan Zhao, Yan Wang, Xinlu Wang, Ruiqing Wang, Na Liu, Peng Li, Min Ji, Jingjing Ye, Tao Sun, Jingxin Li, Daoxin Ma, Fei Lu, Chunyan Ji

**Affiliations:** 1 Department of Hematology, Qilu Hospital of Shandong University, Jinan, 250012 Shandong China; 2 Department of Hematology, Taian central hospital, Taian, 271000 Shandong China; 3 Department of Physiology, School of Basic Medical Sciences, Shandong University, Jinan, 250012 Shandong China

**Keywords:** TNFAIP8, Apoptosis, Chemoresistance, ERK, Rac1, AML

## Abstract

**Background:**

Chemoresistance is emerging as a major barrier to successful treatment in acute myeloid leukemia (AML), and evasion of apoptosis is among the fundamental underlying mechanisms. Therefore, unraveling molecular networks that drive this process constitutes an urgent unmet need. Herein, we aim to characterize the role and molecular mechanism of the tumor necrosis factor ɑ-induced protein 8 (TNFAIP8), a novel anti-apoptotic molecule, in AML chemoresistance.

**Methods:**

The expression levels of TNFAIP8 were assessed in AML patients and cell lines by RT-qPCR and western blots. The transcriptional regulation of TNFAIP8 was analyzed with luciferase reporter assay and ChIP followed by RT-qPCR. Functional experiments were conducted to evaluate the effects of TNFAIP8 on apoptosis, drug sensitivity and proliferation of AML cells. Potential effects of TNFAIP8 on the activation of extracellular signal-regulated kinase (ERK) pathway were detected by western blots. CoIP and P21-activated kinase (PAK) pull-down assay were performed to ascertain the upstream target. The overall effects of TNFAIP8 on AML were examined in murine models.

**Results:**

Upregulated TNFAIP8 expression was first confirmed in human AML patients and cell lines. E74 like ETS transcription factor 1 (ELF1) was then identified to contribute to its aberrant expression. Through manipulating TNFAIP8 expression, we described its role in protecting AML cells from apoptosis induced by chemotherapeutic agents and in promoting drug resistance. Notably, the leukemia-promoting action of TNFAIP8 was mediated by sustaining activity of the ERK signaling pathway, through an interaction with Rac family small GTPase 1 (Rac1). In addition, in vivo experiments confirmed that TNFAIP8 suppression lowered leukemia infiltration and improved survival.

**Conclusion:**

Our data provide a molecular basis for the role of TNFAIP8 in chemoresistance and progression of AML and highlight the unique function of TNFAIP8 as an attractive therapeutic target.

## Background

Acute myeloid leukemia (AML), a rapidly progressing hematopoietic malignancy, is the most common form of acute leukemia in adults [1]. Nowadays, extensive use of standard therapy induces complete remission (CR) in approximately 50 to 70% of AML patients, but about 76% of patients relapse or die eventually [2–5], indicating that drug resistance has become a major obstacle to an optimistic prognosis. Therefore, it is of vital importance to explore the molecular rationale underlying drug resistance in AML.

Apoptosis induction underlies the therapeutic effects of most conventional antineoplastic agents. However, an expanding body of evidence has illustrated that dysregulated expression of anti-apoptotic molecules, such as BCL-2 and MCL-1, renders malignant cells resistant to the pro-apoptotic effects of cytotoxic agents [6–8]. Inhibition of these molecules has increased chemosensitivity in AML, underlining the crucial role of apoptosis dysregulation in AML chemoresistance [9–11]. Nevertheless, resistance to the currently available targeted inhibitors has also emerged afterwards, suggesting inter-complementary effects among anti-apoptotic molecules [12–16]. Thus there arise demands for further exploration of new candidates in apoptosis regulation network in AML.

Tumor necrosis factor α-induced protein 8 (TNFAIP8), also called SCC-S2, GG2-1, MDC-3.13, NDED and OXI-α, containing a death effector domain, was first found in human head and neck squamous cell carcinoma [17]. Its aberrant expression has been successively validated in cancers of breast, esophagus, lung, ovary, stomach and others [17–25]. Subsequent research provided insight into the regulatory function of TNFAIP8 in cell apoptotic network. TNFAIP8 is capable of suppressing apoptosis by inhibiting caspase activation, thus promoting cisplatin resistance in cervical carcinoma [24]. In addition, TNFAIP8 has been reported to promote p53 ubiquitination and decrease p53-dependent pro-apoptotic responses, promoting drug resistance to cisplatin and doxorubicin of NSCLC cells, respectively [21, 23]. Moreover, TNFAIP8 has been found to be highly expressed in AML and acute lymphoblastic leukemia cell lines [18]. However, detailed roles and mechanisms of TNFAIP8 in AML remain unclear.

In this study, we sought to investigate the role and molecular basis of TNFAIP8 in AML chemoresistance. We found that TNFAIP8 reduced cell apoptosis and increased chemoresistance in vitro. Mechanistically, we identified that ELF1 served as a positive regulator of TNFAIP8 transcription. And TNFAIP8 was found to enhance activity of ERK signaling pathway through interaction with Rac1, contributing to its anti-apoptotic effects on AML cells. Finally, by using a murine leukemia model, we found mice bearing murine AML cells with TNFAIP8 inhibition showed improved survival. Collectively, our data demonstrate that TNFAIP8 promotes chemoresistance and progression in AML and that targeting TNFAIP8 may be a promising strategy for AML treatment.

## Methods

### Patient samples

Bone marrow (BM) samples from 60 AML patients and 17 control donors were obtained at the Qilu Hospital of Shandong University, China. Samples were collected from AML patients at different stages of the disease, including patients with newly diagnosed AML (*n* = 28), patients with relapsed/refractory AML (*n* = 13) and patients with complete remission (*n* = 19). Control samples were obtained from donors without any malignant bone marrow disorder. Informed consent was obtained in accordance with the Declaration of Helsinki. All laboratory experiments with primary samples were approved by the Medical Ethics Committee of Qilu Hospital of Shandong University.

### Cell lines and cell culture

Human leukemic cell lines THP1, U937, K562, K562/A02, K562/G01, HL60, HL60/ADR cells and human embryonic kidney 293 T cells were purchased from the Institute of Hematology and Blood Diseases Hospital, Chinese Academy of Medical Sciences and Peking Union Medical College, Tianjin, China. Murine leukemic cell line C1498 was purchased from ATCC. THP1, U937, K562, K562/A02, K562/G01 cells were cultured in RPMI 1640 medium, HL60 and HL60/ADR cells were cultured in IMDM medium, 293 T cells were cultured in DMEM medium (with 10% heat-inactivated fetal calf serum, Gibco; penicillin and streptomycin, Invitrogen; 37 °C, 5% CO_2_, in humidified incubator). Doxorubicin was added (final concentration of 0.5 μg/mL) to the complete culture medium of K562/A02 and HL60/ADR until 2 weeks before experiments. Multidrug resistant cell lines, HL60/ADR [26] and K562/A02 [27], and parental HL60 and K562 cell lines were used to investigate the effect of TNFAIP8 levels on chemoresistance in vitro. Cell line identity and purity were verified regularly by short tandem repeat profiling. The latest authentication of cell lines was conducted by Shanghai Zhong Qiao Xin Zhou Biotechnology Co., Ltd. (*July* 16 to *August* 23*, 2019*).

### Chemical inhibitors

ERK1/2 inhibitor SCH772984 (MedChemExpress) was dissolved in DMSO (2.5 μM in culture). Rac1 inhibitor EHOP-016 trihydrochloride (Selleck) was dissolved in DMSO (5 μM in culture).

### Lentiviral transduction

Lentiviral constructs repressing TNFAIP8, expressing TNFAIP8 with Flag-tagged or expressing ELF1 were purchased from Genechem (Shanghai, China). Those repressing mouse TNFAIP8 were also purchased from Genechem (Shanghai, China) and were used to establish C1498 cell line constitutively repressing TNFAIP8 (Table 1). Cells were infected with lentivirus for 24 h and selected by puromycin.

**Table 1:** Target sequence for TNFAIP8 shRNAs

shRNAs	sequence (5′ to 3′)
TNFAIP8 (homo) shRNA	TTGGATGAAGAGAACATAT
Scrambled (homo) control	TTCTCCGAACGTGTCACGT
Tnfaip8 (mus) shRNA1	GCTGCCTTGTACAATCCCTTT
Tnfaip8 (mus) shRNA2	CCACAGGAACAATCAGTTCAA
Scrambled (mus) control	TTCTCCGAACGTGTCACGT

### Quantitative reverse transcription PCR (RT-qPCR)

Total RNA was extracted using TRIzol (Invitrogen). Reverse transcription was performed with a M-MLV RTase cDNA Synthesis Kit (Takara, Japan). RT-qPCR was performed by an Applied Biosystem 7900HT System (ABI) with appropriate primers (Table 2), SYBR Green PCR Master Mix (Toyobo, Japan), and GAPDH or ACTB as internal controls. Each sample was amplified in a 10 μL reaction volume according to manufacturer’s instructions.

**Table 2:** RT-qPCR primers

Primer Name	Sequence (5′ to 3′)
TNFAIP8 (homo)-Forward	GCCGTTCAGGCACAAAAGA
TNFAIP8 (homo)-Reverse	GCACCTCACTACTTGTGTCGTCTATT
ELF1 (homo)-Forward	AGAGTCTTCAGATCCATCGCTA
ELF1 (homo)-Reverse	GGTTTTGCAGCTTTAGAATTCCC
GAPDH (homo)-Forward	GGAGCGAGATCCCTCCAAAAT
GAPDH (homo)-Reverse	GGCTGTTGTCATACTTCTCATGG
Tnfaip8 (mus)-Forward	GGTATCCAAATCCATCGCCACCA
Tnfaip8 (mus)-Reverse	CCAGCTCGTCTTGATTGAACTGA
ACTB (mus)-Forward	TACTGAGCTGCGTTTTACACC
ACTB (mus)-Reverse	TCCTGAGTCAAAAGCGCCAA

### Western blot

Cells were lysed in a protein solubilization buffer. Protein extracts were prepared with a Total Protein Extraction Kit according to the manufacturer’s instructions (BestBio, Shanghai, China). Proteins were separated by SDS-PAGE and transferred to nitrocellulose membranes (Millipore). β-actin or GAPDH served as a loading control. Primary antibodies included anti-GAPDH, anti-β-actin (ZSGB-BIO, China), anti-TNFAIP8, anti-Rac1 (Proteintech), anti-Flag (Sigma), as well as anti-ERK1/2, anti-p-ERK1/2, anti-MEK, and anti-p-MEK (CST). Protein bands were visualized using a FluorChem E Chemiluminescent Western Blot Imaging System (Cell Biosciences).

### Proliferation and IC_50_

To assess proliferation, cells were plated in 96-well plates and cultured in an incubator at 37 °C. At each time point for the next 3 days, 10 μL CCK-8 (BestBio, Shanghai, China) was added to each well, then cells were incubated for 4 h. Absorbance (450 nm) was measured by a Microplate Reader (Thermo Scientific).

To measure half maximal inhibitory concentration (IC_50_), cells were exposed to serial dilutions of doxorubicin (ADM), cytarabine (Ara-C) or idarubicin (IDA) (Sigma) for 48 h. Cell viability was determined by CCK-8 assays and IC_50_ values were calculated.

### Apoptosis

Cells were treated with doxorubicin, cytarabine or idarubicin for 48 h, stained with Annexin V/PI (BestBio, Shanghai, China) or Annexin V/7-AAD (BestBio, Shanghai, China), and apoptosis was analyzed by flow cytometry (Beckman Coulter).

### Luciferase assay

Twenty-four hours before transfection, cells were plated in 24-well dishes at 5 × 10^4^ cells/well. Transfections included a constant amount of Renilla luciferase plasmid for internal control. Fourty-eight hours after transfection, cells were incubated with passive lysis buffer (15 min, 160 μL/well, Promega), then 25 μL of each lysate was subjected to a dual luciferase assay (Promega). Luciferase activity was measured using a luminometer (LB 96v, Berthold). Results of triplicate transfections were normalized to Renilla luciferase activity.

### Chromatin immunoprecipitation (ChIP)

The SimpleChIP® Enzymatic Chromatin IP Kit (Cell Signaling Technology) was used to perform ChIP assays according to the manufacturer’s protocol. Chromatin fragments derived from K562, K562/A02, HL60 and HL60/ADR cells were immunoprecipitated with 5 μg ELF1 antibody (Proteintech). The 5′-upstream region of human TNFAIP8 gene (− 1154 to − 1142, TNFAIP8-promoter) was obtained by PCR of genomic DNA using the following primers: forward 5′- TTCTTCCAAACCCAGCTCAGAC - 3′; reverse 5′- AAACATACACAAGGTACGGAGG - 3′.

### Co-immunoprecipitation (CoIP)

Total protein was incubated with anti-Flag or anti IgG antibodies (16 h, 4 °C, rotation), then protein A/G PLUS-agarose beads were added (20 μL, 1 h, 4 °C, rotation; Santa Cruz Biotechnology). Captured agarose beads-Ab-Ag complexes were washed (five times, PBS) and detected by western blot.

### P21-activated kinase (PAK) pull-down

K562, HL60, K562/A02 and HL60/ADR cells were serum-starved (16 h), then treated with doxorubicin (4 h, 0.5 μg/mL for K562 and HL60, 5 μg/mL for K562/A02 and HL60/ADR). A Rac1 activation assay biochem kit (Cytoskeleton) was used to perform PAK pull-down assays according to the manufacturer’s protocol.

### In vivo model of AML

Animal studies were conducted in compliance with institutional guidelines and were approved by the Medical Ethics Committee of Qilu Hospital of Shandong University. Female C57BL/6 mice (20; 8-week-old; Jinan pengyue laboratory animal breeding co., Ltd., Jinan, China) were intravenously injected with C1498 cells (2 × 10^5^ cells/100 μL) transduced with a non-targeted short hairpin RNA (C1498: shNC, *n* = 10 mice) or TNFAIP8 shRNA (C1498: shTNFAIP8, n = 10 mice). Mice were monitored daily for evidence of leukemia.

On day 24, mice were euthanized and livers and spleens were measured. Leukemia infiltration into peripheral blood, bone marrow, spleen and liver was evaluated by flow cytometry detection of GFP-positive cells and hematoxylin and eosin staining. Liver, spleen and bone marrow tissues of mice were fixed (10% formaldehyde), paraffin-embedded, sectioned (4 μm), and stained with hematoxylin and eosin. Immunohistochemical detection of TNFAIP8 and p-ERK1/2 (CST) was performed on bone marrow samples. After heat-induced epitope retrieval, bone marrow samples were incubated with primary antibodies (4 °C, overnight), then with secondary antibody in a biotin-streptavidin HRP detection system, and finally 3,3′-diaminobenzidine was used for detection and visualization. Slides were observed by a microscope (Nikon, Ni-U). Survival was also followed and presented with a Kaplan-Meier survival plot.

### Statistical analyses

Data are presented as mean ± standard deviation (SD). Differences between 2 groups were analyzed using an unpaired Student t-test. Differences between 3 or 4 groups were analyzed using one-way ANOVA or two-way ANOVA followed by LSD test with normally distributed data. The Mann-Whitney U test was used for cases with unequal variances. Survival was presented with a Kaplan-Meier survival plot. *P* < 0.05 was considered statistically significant.

## Results

### TNFAIP8 is highly expressed in primary AML samples and cell lines

**Fig. 1:**
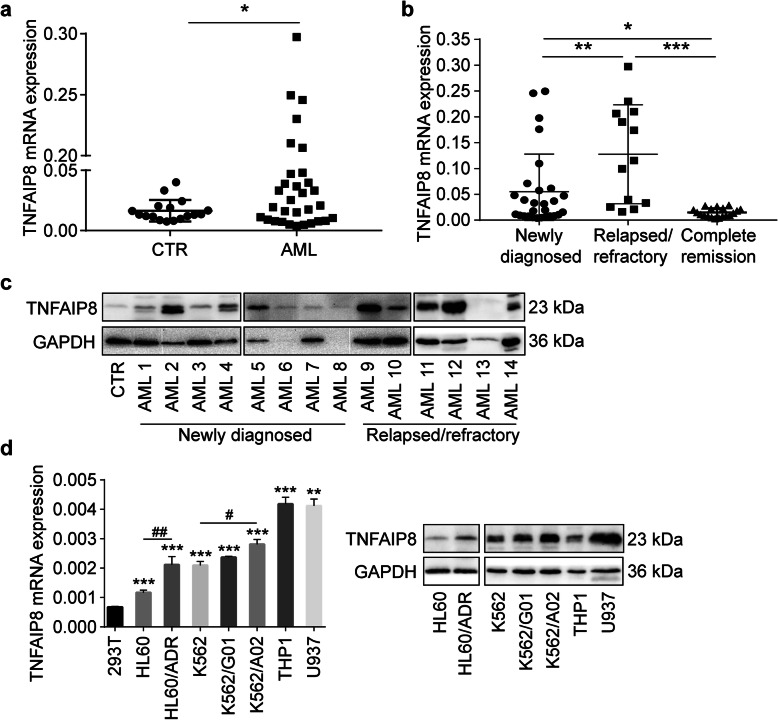
Expression of TNFAIP8 in primary AML samples and cell lines. **a** Quantitative mRNA expression of TNFAIP8 by RT-qPCR in AML patient samples (*n* = 41) and controls (CTR, *n* = 17). **b** Quantitative mRNA expression of TNFAIP8 in patients with newly-diagnosed AML (n = 28), patients with relapsed/refractory AML (*n* = 13) and patients with complete remission (n = 19). Data are mean ± SD values calculated by Mann-Whitney U test. * *P* < 0.05; ** *P* < 0.01. **c** Representative immunoblotting analysis of TNFAIP8 in patients with newly-diagnosed AML (*n* = 8) and patients with relapsed/refractory AML (*n* = 6) relative to control (CTR, n = 1). **d** RT-qPCR and immunoblotting analysis of TNFAIP8 expression in acute myeloid leukemia cell lines relative to 293 T cell line (repeated three times). Data are mean ± SD values calculated by Mann-Whitney U test or one-way ANOVA. * *P* < 0.05, ** *P* < 0.01, *** *P* < 0.001 versus the 293 T group; # *P* < 0.05, ## *P* < 0.01 means the drug resistant cell line (K562/A02 or HL60/ADR) versus the sensitive cell line (K562 or HL60)

TNFAIP8 mRNA levels were determined in AML patient bone marrow samples (*n* = 41) and control samples (*n* = 17). Clinical characteristics are summarized in Table 3. TNFAIP8 expression was significantly increased in AML compared to control (*P* < 0.05, Fig. 1a). TNFAIP8 expression was higher in patients with newly diagnosed AML (*n* = 28, *P* < 0.05) and patients with relapsed/refractory AML (*n* = 13, *P* < 0.001) than in patients with complete remission (*n* = 19, Fig. 1b). Besides, patients with relapsed/refractory AML exhibited the highest level (newly-diagnosed vs relapsed/refractory, *P* < 0.01, Fig. 1b). Compared to control, TNFAIP8 protein is generally expressed in AML patients (Fig. 1c; Additional file 2: Figure S1). These data suggested that TNFAIP8 expression could be related to therapeutic efficacy.

**Table 3:** Clinical characteristics of AML patients

Characteristic	Newly diagnosed (***n*** = 28)	Relapsed/refractory (***n*** = 13)	Complete remission (***n*** = 19)
**Age, y**			
Median	52	53	42
Range	13–78	18–69	18–68
**Male, n (%)**	16 (28)	7 (13)	11 (19)
**WBC, × 10**^9^**/L**			
Median	7.14	2.73	4.64
Range	1.2–263.1	1.2–198.4	1.05–21.56
**HGB, g/L**			
Median	79	79	118
Range	3–135	32–109	66–144
**PLT, × 10**^9^**/L**			
Median	39	47	174
Range	5–569	8–357	128–338
**Blast in bone marrow, %**			
Median	77.56	44	1.57
Range	13.63–97.00	10–91	0.36–3.15
**FAB classification**			
M0	0	0	1
M1	2	1	0
M2	3	2	2
M3	0	0	3
M4	1	0	3
M5	14	9	10
M6	0	0	0
M7	0	0	0
Unclassified	8	1	0
**Cytogenetics, n (%)**			
Normal	11	6	15
Abnormal	13	6	2
Unknown	4	1	2

*AML* acute myeloid leukemia, *HGB* hemoglobin, *PLT* platelets, *WBC* white blood cells, *FAB* French-American-British classification

We then evaluated TNFAIP8 expression in leukemia cell lines. TNFAIP8 is universally expressed in 7 AML cell lines compared with 293 T cells (Fig. 1d). Among AML cell lines, parental sensitive AML cell lines, K562 and HL60, showed lower levels of TNFAIP8 than corresponding chemoresistant AML cell lines, K562/A02 and HL60/ADR (K562 vs K562/A02, *P* < 0.05; HL60 vs HL60/ADR, *P* < 0.01; Fig. 1d). Thus, TNFAIP8 may play an important role in AML drug resistance.

### ELF1 promotes human *TNFAIP8* gene transcription in AML

**Fig. 2:**
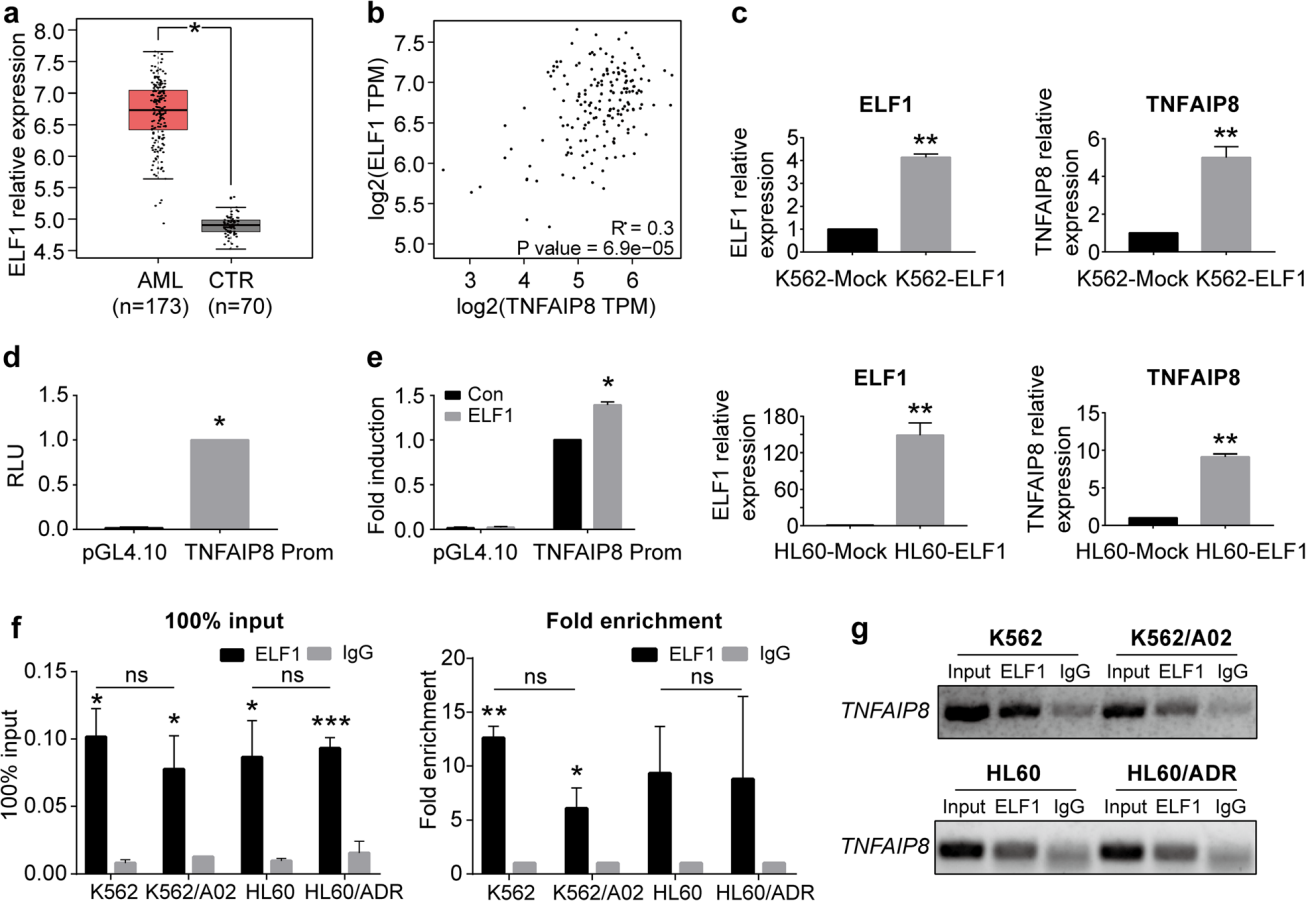
ELF1 promotes human *TNFAIP8* gene transcription in AML. **a** ELF1 expression in AML patient samples (*n* = 173, red) and control BM samples (*n* = 70, grey) from TCGA database. **b** Correlation of TNFAIP8 and ELF1 expression (Log2 transformed values; Pearson correlation, R) in AML patients from TCGA database. **c** TNFAIP8 and ELF1 expression in K562 and HL60 cells infected with lentivirus harboring ELF1 (ELF1) or negative control (Mock) by RT-qPCR. Data are mean ± SD values of three independent experiments. * *P* < 0.05; ** *P* < 0.01; *** *P* < 0.001. **d** The putative promoter of the TNFAIP8 was cloned upstream of firefly luciferase to yield plasmid TNFAIP8-Prom. TNFAIP8-Prom or empty pGL4.10 vector was transfected into 293 T cells. Dual-luciferase activity was measured 48 h after transfection by a luminometer. Luciferase activity was normalized for Renilla luciferase. Results are represented as mean ± SD and calculated as fold induction relative to cells transfected with TNFAIP8-Prom plasmid. **e** 293 T cells were transfected with the indicated luciferase reporter plasmids, together with ELF1 expression plasmid. Results are represented as mean ± SD and calculated as fold induction relative to cells transfected with TNFAIP8-Prom plasmid and control blank vector of ELF1. **f, g** ChIP analysis of ELF1 binding to *TNFAIP8* promoter region. Input served as a positive control and IgG IP was used as a negative control for ChIP. The fold enrichment values were normalized to the negative control IgG. Data are mean ± SD values of three independent experiments calculated by Mann-Whitney U test or unpaired Student t-test. * *P* < 0.05; ** *P* < 0.01; *** *P* < 0.001 versus the IgG group, ns = not significant

To analyze the potential regulatory mechanism of TNFAIP8 expression, we took advantage of CHIPBase to search for potential DNA-binding proteins that could bind the sequence of the *TNFAIP8* promoter [28]. Among these proteins, ELF1 possesses the most potential binding sites (Additional file 1: Table S1). *ELF1* was significantly elevated in AML patients compared with controls (Fig. 2a) using TCGA data [29]. Besides, a positive correlation was observed between ELF1 and TNFAIP8 expression in AML patients (Fig. 2b). In AML cell lines, parental sensitive AML cell lines, K562 and HL60, showed lower levels of ELF1 than corresponding chemoresistant AML cell lines, K562/A02 and HL60/ADR (K562 vs K562/A02, *P* < 0.05; HL60 vs HL60/ADR, *P* < 0.05; Additional file 3: Figure S2a). To investigate whether ELF1 is functionally involved in regulation of TNFAIP8 expression, we detected TNFAIP8 expression followed by ELF1 overexpression in K562 and HL60 cell lines, and found that upregulation of ELF1 increased TNFAIP8 expression levels (*P* < 0.01; Fig. 2c). We then detected TNFAIP8 expression followed by ELF1 knockdown in K562/A02 and HL60/ADR cell lines, and found that downregulation of ELF1 decreased TNFAIP8 expression levels (Additional file 3: Fig. S2b).

To evaluate the ability of ELF1 to confer transcriptional activation of TNFAIP8, a region of genomic DNA comprising the 1.3 kb fragment located in the 5′-flanking region of the human *TNFAIP8* gene was cloned in front of the firefly luciferase gene in the vector pGL4.10. The resultant plasmid (TNFAIP8-Prom) was transfected into 293 T cells and luciferase activity was measured by a luminometer to reflect TNFAIP8 promoter activity. As shown in Fig. 2d, TNFAIP8-Prom plasmid-transfected cells had significantly higher luciferase activity compared with controls, indicating that the 1.3-kb fragment contains the functional promoter region of the human *TNFAIP8* gene. Then we co-transfected 293 T cells with ELF1 expression plasmid and TNFAIP8-Prom plasmid and found that overexpression of ELF1 caused an increase in luciferase expression from TNFAIP8-Prom (Fig. 2e). Thus these data support a role for ELF1 in transcriptional regulation of TNFAIP8.

To identify the functional site of ELF1 in the *TNFIAP8* gene promoter, ChIP was used to pull down the ELF1-bound DNA. We found significant enrichment of a sequence (− 1154 to − 1142 bp of *TNFAIP8* promoter) in ELF1 immunoprecipitate compared with IgG immunoprecipitate (Fig. 2f, right). The percent of ELF1 group relative to the input was higher than the negative background IgG group (Fig. 2f, left). No significant difference was found in fold enrichment or percentage of input between resistant and sensitive AML cell lines. Agarose gel electrophoresis (AGE) analysis showed that ELF1 antibody effectively immunoprecipitated the sequence from − 1154 to − 1142 bp of *TNFAIP8* promoter (Fig. 2g). These data indicated that the site from − 1154 to − 1142 bp of the *TNFAIP8* promoter was essential for ELF1 regulation. Taken together, ELF1 is recruited to the *TNFAIP8* promoter, thereby facilitating transcription of TNFAIP8.

### TNFAIP8 suppression inhibits cell growth, enhances chemosensitivity and apoptosis induced by chemotherapeutics

**Fig. 3:**
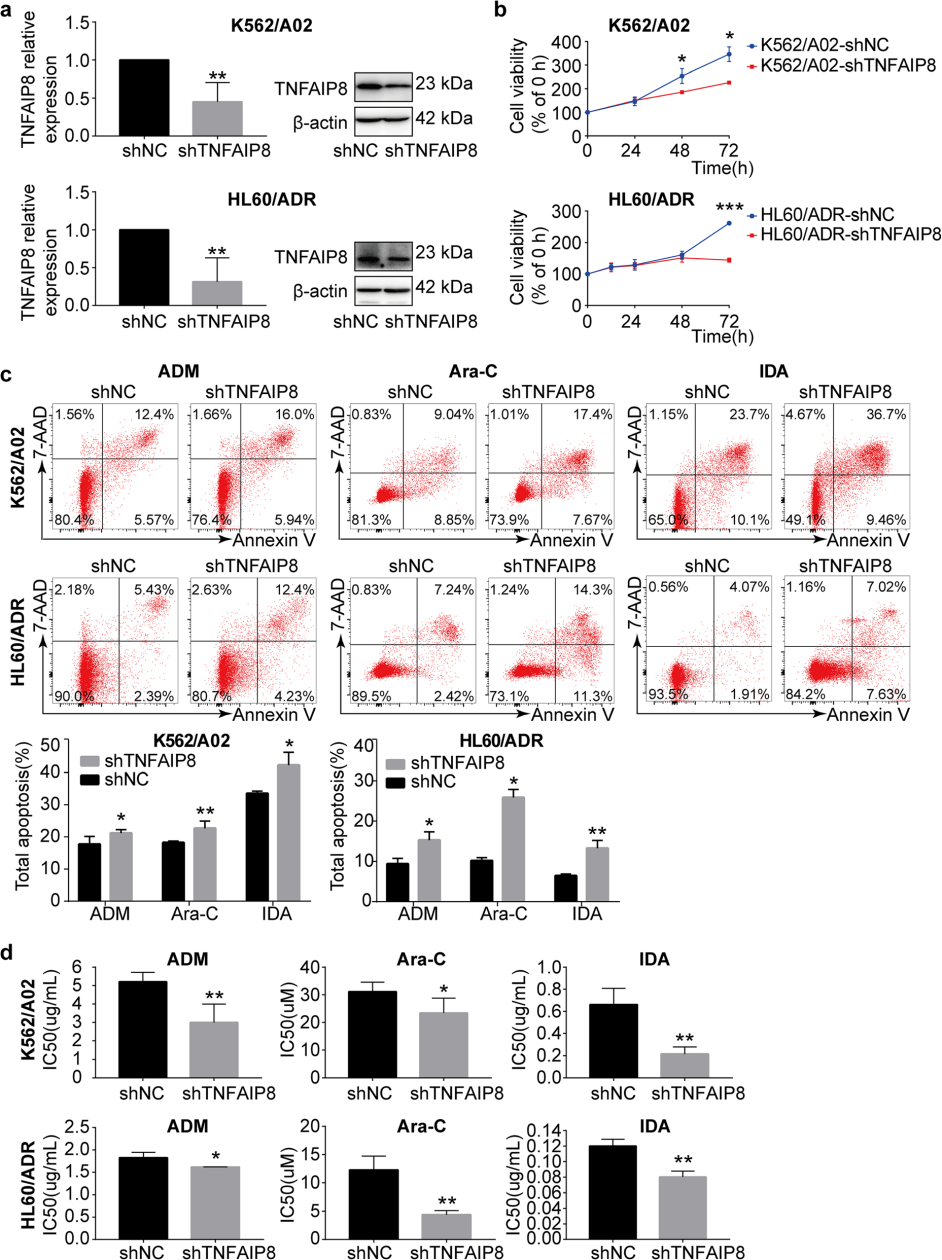
TNFAIP8 suppression inhibits cell growth and enhances chemosensitivity and apoptosis in chemoresistant cell lines K562/A02 and HL60/ADR. **a** TNFAIP8 knockdown (shTNFAIP8) or nonsilencing scrambled control (shNC) K562/A02 and HL60/ADR cells were selected by puromycin followed by RT-qPCR and western blots with indicated antibodies. **b** Proliferation of K562/A02 cells (shTNFAIP8 or shNC) and HL60/ADR cells (shTNFAIP8 or shNC) were assessed by CCK8 assays, and proliferation rates at 0, 12, 24, 48 and 72 h were calculated normalized to the absorbance at 0 h. **c** K562/A02 (shTNFAIP8 or shNC) and HL60/ADR cells (shTNFAIP8 or shNC) were treated with ADM (20 μg/mL), Ara-C (30 μM) and IDA (0.2 μg/mL) for 48 h to measure apoptosis by flow cytometry. **d** IC_50_ values of K562/A02 cells (shTNFAIP8 or shNC) and HL60/ADR cells (shTNFAIP8 or shNC) were calculated according to cell growth inhibition after 48 h treatment with serial dilutions of ADM, Ara-C and IDA. Data are mean ± SD values of three independent experiments calculated by Mann-Whitney U test or unpaired Student t-test. * *P* < 0.05; ** *P* < 0.01; *** *P* < 0.001

To explore the functional significance of TNFAIP8 in leukemia drug resistance, we downregulated TNFAIP8 expression in K562/A02 and HL60/ADR cells by RNAi. Suppression of TNFAIP8 was verified by RT-qPCR and western blot (Fig. 3a). TNFAIP8 downregulation significantly inhibited cell growth (Fig. 3b). Additionally, apoptosis induced by chemotherapeutics was increased after TNFAIP8 knockdown (Fig. 3c). Similarly, TNFAIP8 knockdown reduced the IC_50_ of chemotherapeutics in K562/A02 and HL60/ADR, confirming that TNFAIP8 ablation can re-sensitize AML-resistant cells to chemotherapeutics, including doxorubicin, cytarabine and idarubicin (Fig. 3d). The function of TNFAIP8 was further detected in another two hematological malignant cell lines, THP1 and U937 (Additional file 5: Figure S4) [30, 31]. We then examined the effects of TNFAIP8 knockdown on caspase activation. Increased activation of caspase 3 and caspase 8, as expected, were observed after TNFAIP8 knockdown in HL60/ADR cells and K562/A02 cells (Additional file 4: Figure S3b, S3d). Thus, TNFAIP8 is important for regulation of apoptosis induced by chemotherapy and chemoresistance, as well as for maintenance of cell proliferative potential in AML.

### TNFAIP8 overexpression promotes cell proliferation and drug resistance, and protects cells from apoptosis induced by chemotherapeutics

**Fig. 4:**
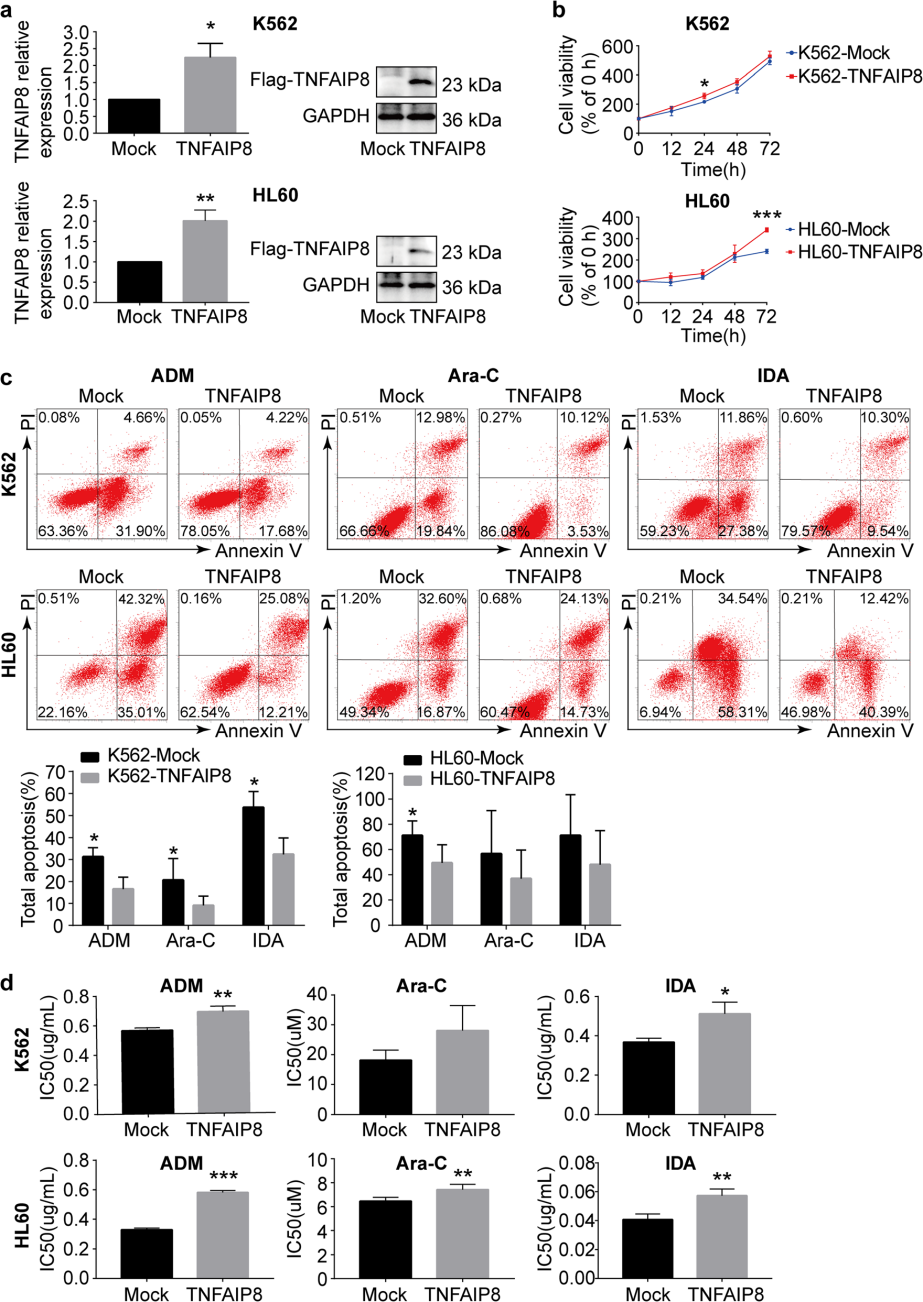
TNFAIP8 overexpression promotes cell proliferation and drug resistance and decreases apoptosis in chemosensitive cell lines K562 and HL60. **a** K562 and HL60 were transduced with Flag-tagged TNFAIP8 (TNFAIP8) or control lentivirus (Mock). Cells were sorted by puromycin followed by quantitative PCR and western blots with indicated antibodies. **b** Proliferation of K562 cells (TNFAIP8 or Mock) and HL60 cells (TNFAIP8 or Mock) was assessed by CCK8 assays, and proliferation rates at 0, 12, 24, 48 and 72 h were calculated normalized to the absorbance at 0 h. **c** K562 cells (TNFAIP8 or Mock) and HL60 cells (TNFAIP8 or Mock) were treated with ADM (2 μg/mL), Ara-C (10 μM) and IDA (0.05 μg/mL) for 48 h to measure apoptosis by flow cytometry. **d** IC_50_ values of K562 cells (TNFAIP8 or Mock) and HL60 cells (TNFAIP8 or Mock) were calculated according to cell growth inhibition after 48 h treatment with serial dilutions of ADM, Ara-C and IDA. Data are mean ± SD values of three independent experiments calculated by Mann-Whitney U test or unpaired Student t-test. * *P* < 0.05; ** *P* < 0.01; *** *P* < 0.001

We then investigated the effects of TNFAIP8 overexpression on cell growth and chemotherapy treatment. We used a lentivirus vector to overexpress TNFAIP8 in AML cells. Infection with TNFAIP8-expressing lentivirus increased TNFAIP8 expression effectively (Fig. 4a; Additional file 6: Figure S5a). TNFAIP8 overexpression increased cell proliferation (Fig. 4b; Additional file 6: Figure S5b). Additionally, apoptotic cell death induced by chemotherapeutics was reduced after TNFAIP8 overexpression in K562, HL60 and THP1 (Fig. 4c; Additional file 6: Figure S5c). Likewise, TNFAIP8 upregulation increased the IC_50_ of chemotherapeutics (Fig. 4d). The effect of TNFAIP8 overexpression on caspase activation was also measured. Decreased activation of caspase 3 and caspase 8 were observed after TNFAIP8 overexpression in HL60 cells and K562 cells (Additional file 4: Figure S3a, S3c). These findings confirmed that TNFAIP8 plays an important role in regulation of cell proliferation, apoptosis and drug resistance in AML.

### TNFAIP8 promotes anti-apoptotic phenotype of AML cells in an ERK-dependent manner under pressure of chemotherapeutics

We subsequently explored mechanisms underlying functions of TNFAIP8 in AML chemoresistance. With RNA-sequencing, we found that TNFAIP8 suppression in AML cells under the pressure of chemotherapeutics dramatically changed expression of genes associated with cell proliferation, apoptosis and oncogenesis (Additional file 8: Figure S7). Since the ERK pathway is pivotal for regulating these processes in AML [32–39], we speculated that TNFAIP8 knockdown altered the gene expression profile by influencing ERK signaling.

**Fig. 5:**
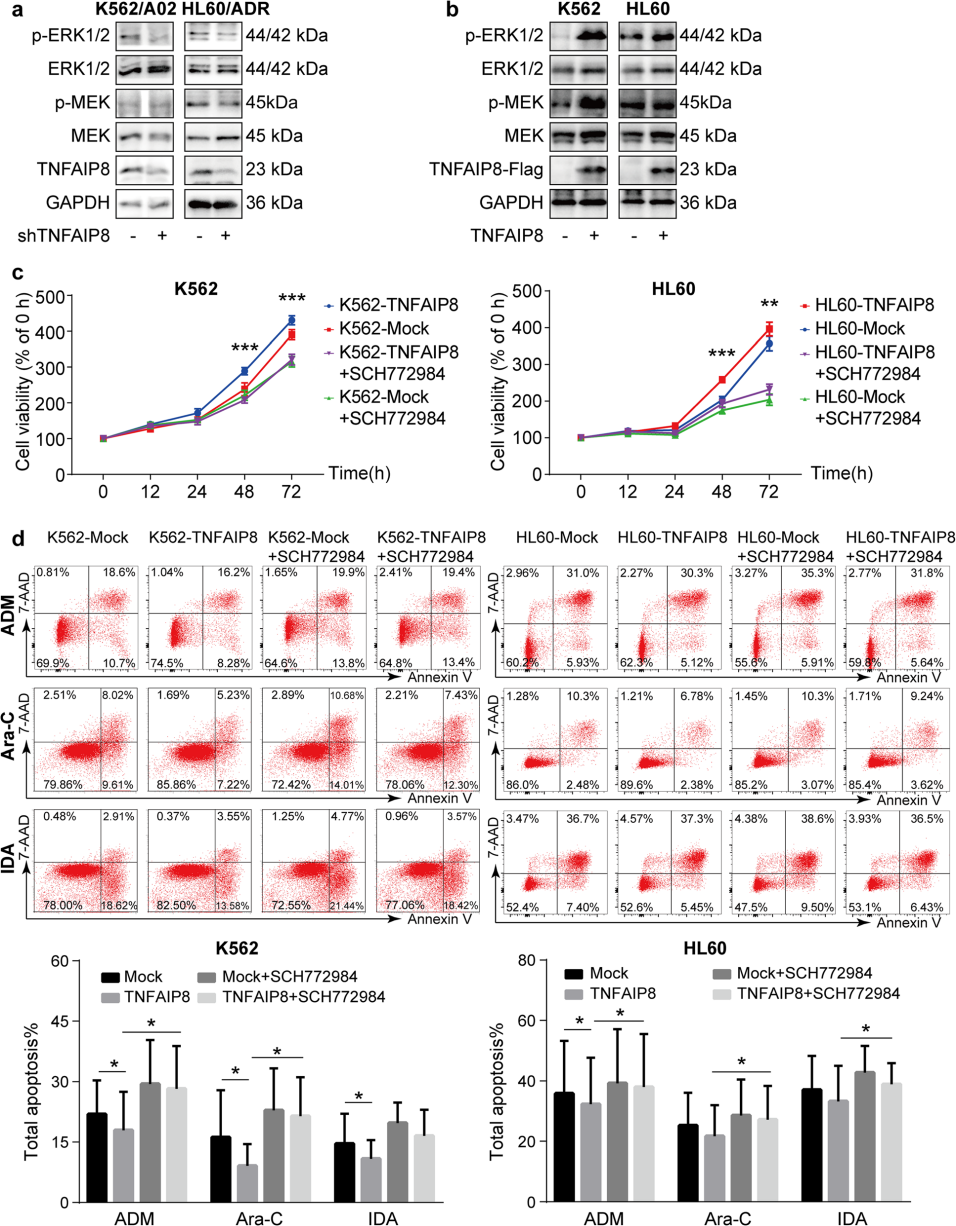
TNFAIP8 promotes cell proliferation and protects cell from apoptosis induced by chemotherapeutics in an ERK-dependent manner. **a, b** Representative immunoblotting of phosphorylated proteins of MAPK signaling cascades in K562, HL60 cells transduced with Flag-tagged TNFAIP8 or control vector, and corresponding resistant cell lines K562/A02 and HL60/ADR transduced with shTNFAIP8 or shNC*.* Cells were stimulated with doxorubicin (1 μg/mL) for 4 h. The experiment was repeated three times with similar results. **c, d** AML cells, K562 and HL60, transduced with Flag-tagged TNFAIP8 or control vector were treated with ERK inhibitor SCH772984 (2.5 μM). Cell proliferation was measured by CCK8 and apoptosis induced by chemotherapeutic agents was measured by flow cytometry. Data are mean ± SD values of four independent experiments calculated by two-way ANOVA followed by LSD. * *P* < 0.05; ** *P* < 0.01; *** *P* < 0.001

Prior to proposing this hypothesis, we evaluated the influence of doxorubicin-treating time on ERK1/2 activation and found a maximum increase of the phosphorylation of ERK1/2 8 h after doxorubicin treatment (Additional file 7: Figure S6a). Immunoblotting analysis confirmed that TNFAIP8 knockdown suppressed phosphorylation of ERK and MEK in AML chemoresistant cells after 8-h treatment of doxorubicin, while TNFAIP8 overexpression increased ERK and MEK phosphorylation in AML chemosensitive cells (Fig. 5a-b). Further, the effect of TNFAIP8 on ERK phosphorylation is independent of the doxorubicin dose (Additional file 7: Figure S6b).

To investigate whether the effect of TNFAIP8 on AML cells is mediated by ERK signaling, we treated AML cells, K562 and HL60, transduced with Flag-tagged TNFAIP8 or control vector with ERK inhibitor SCH772984, and examined their proliferation and chemotherapeutics-aroused apoptosis. We observed that ERK inhibition reversed growth advantage of TNFAIP8-transduced cells (Fig. 5c). In addition, we found that the difference between the Mock group and TNFAIP8 group without ERK inhibitor was statistically significant in the presence of doxorubicin or cytarabine (Mock vs TNFAIP8, *P < 0.05, Fig. 5d), while the difference between the Mock group and TNFAIP8 group with ERK inhibitor was not statistically significant. In other words, the ERK inhibition partially abrogated the down-regulation on the apoptotic level by overexpressing TNFAIP8. Together, these results indicate that TNFAIP8 may affect proliferative capacity and chemotherapy-induced apoptosis partially in an ERK-dependent manner.

### TNFAIP8 promotes ERK activation by interacting with Rac1, a MAPK upstream factor

To investigate how TNFAIP8 regulates ERK signaling pathway, we sought potential interacting partners of TNFAIP8 in AML cells. Since Rac1 activation can boost activity of the ERK pathway [40–45], we hypothesized that TNFAIP8 promotes ERK phosphorylation by affecting Rac1.

**Fig. 6:**
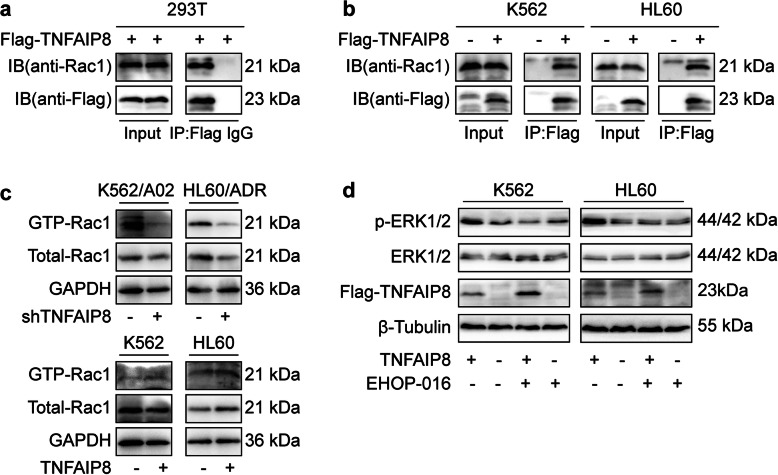
TNFAIP8 promotes ERK activation by interacting with its upstream factor Rac1. **a, b** The interaction of TNFAIP8 and Rac1 was assessed by co-immunoprecipitation experiments in 293 T cells and AML cells, K562 and HL60. Immunoprecipitates were generated by anti-Flag antibody and TNFAIP8-bound Rac1 was detected by immunoblotting with anti-Rac1 antibody. **c** The effect of TNFAIP8 on Rac1 activation. The levels of Rac1-GTP were determined by a PAK-GST pull-down assay in K562, HL60 cells transduced with Flag-tagged TNFAIP8 or control vector, and corresponding resistant cell lines K562/A02 and HL60/ADR transduced with shTNFAIP8 or shNC which were all stimulated with ADM for 4 h. **d** Rac1 inhibition by EHOP-016 (5 μM, 12 h) attenuated the positive effect of TNFAIP8 overexpression on ERK phosphorylation

Co-immunoprecipitation experiments were first performed to ascertain whether TNFAIP8 associates with Rac1. We transfected 293 T cells with Flag-tagged TNFAIP8 and immunoprecipitated cell lysates with anti-Flag Ab or control IgG. Subsequent western blot analysis revealed the specific co-precipitation of endogenous Rac1 by anti-Flag Ab (Fig. 6a). Co-immunoprecipitation of Flag-tagged TNFAIP8 and Rac1 was then detected in AML cells (Fig. 6b), further confirming the physical interaction between TNFAIP8 and Rac1 in vitro. To evaluate the effect of TNFAIP8 on Rac1, we performed PAK-GST pull-down assays in AML cells. PAK-GST beads were used to pull down Rac1-GTP, which is routinely used to assay for Rac1 activity. We found that TNFAIP8 knockdown in AML cells reduced levels of GTP-Rac1 and vice versa, indicating that TNFAIP8 promotes Rac1 activation (Fig. 6c).

To test whether the effect of TNFAIP8 on ERK phosphorylation is Rac1 dependent, we treated AML cells transduced with Flag-tagged TNFAIP8 or control vector with Rac1 inhibitor EHOP-016, and then examined ERK activation. We found that increased ERK activation in TNFAIP8-transduced cells was attenuated by Rac1 inhibition (Fig. 6d). Altogether, these data suggest that TNFAIP8 regulates the ERK signaling pathway by interacting with Rac1.

### TNFAIP8 suppression decelerates AML development and progression in vivo

**Fig. 7:**
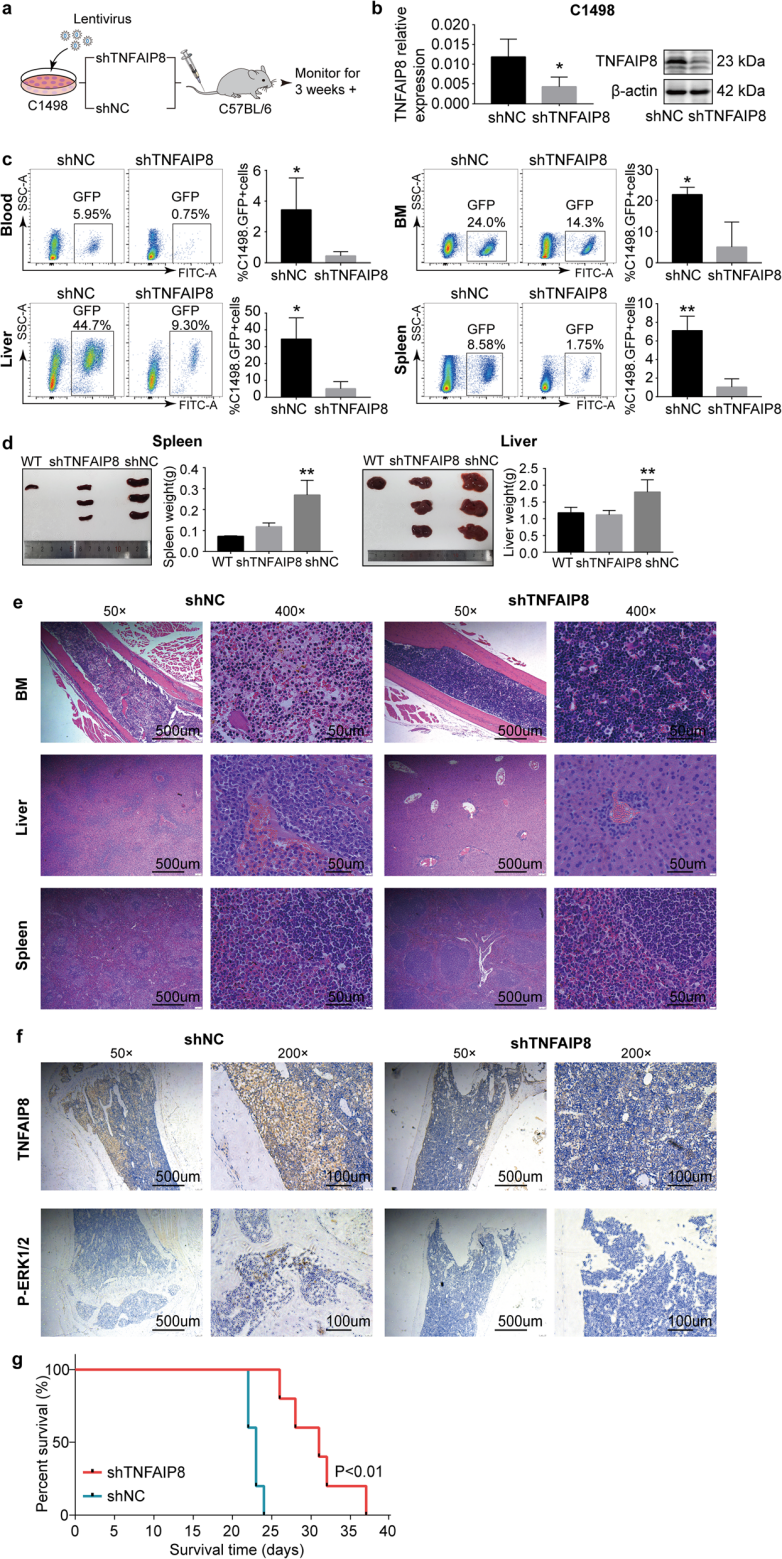
TNFAIP8 suppression decelerates AML progression in vivo. **a** Schematic design of murine AML models. Murine AML cell line C1498 cells was transduced with *Tnfaip8* shRNA (shTNFAIP8) or nonsilencing scrambled control (shNC). C57BL/6 mice were injected with 2 × 10^5^ C1498 cells (shTNFAIP8 or shNC) through tail vein. **b** C1498 cells (shTNFAIP8 or shNC) were sorted for GFP positivity followed by RT-qPCR and western blots. **c** C57BL/6 mice from shTNFAIP8 and shNC group (*n* = 5 in each group) were euthanized to evaluate tumor burden at day 24. Representative flow cytometry plots and summary data of GFP-positive cells rates in blood, bone marrow (BM), liver and spleen. **d** Representative examples and summary weight data of livers and spleens derived from two groups of leukemic mice. **e** Representative Hematoxylin and eosin (H&E) images (original magnification 50× and 400×) from two groups showing different infiltration levels of tumor in blood, bone marrow (BM), liver and spleen. **f** Representative immunohistochemical (IHC) images (original magnification 200×) of BM specimens from two groups showing a difference in TNFAIP8 and p-ERK expression. Data are mean ± SD values. * *P* < 0.05; ** *P* < 0.01; *** *P* < 0.001. **g** For survival analysis, C57BL-6 mice were engrafted with C1498 cells transduced with TNFAIP8 shRNA or nonsilencing scrambled control (n = 5 in each group) and monitored until ethical euthanasia according to signs of morbidity. Survival was plotted by using the Kaplan-Meier method

We examined the in vivo significance of TNFAIP8 in AML using a murine AML model. Murine AML cell line, C1498, was transduced with shRNA targeting mouse *Tnfaip8* or negative control (Fig. 7a). TNFAIP8 suppression in C1498 cells was verified by RT-qPCR and western blot (Fig. 7b). C1498 cells (GFP^+^) were sorted by FACS and injected intravenously into C57BL/6 mice (Fig. 7a), and two weeks later emaciation as well as tetraplegia were observed in two groups, which is indicative of disease onset and progression.

Mice were sacrificed 24 days after injection. Leukemia burden was evaluated by assessing the degree of organomegaly and AML cell infiltration. FACS analysis of peripheral blood, bone marrow, liver and spleen from recipient mice showed reduced circulating GFP^+^ leukemia cells in shTNFAIP8 AML mice (Fig. 7c). Spleen and liver enlargement were significantly less in shTNFAIP8 AML mice (Fig. 7d). H&E analysis confirmed lower leukemia infiltration in the spleen, liver and bone marrow in shTNFAIP8 AML mice (Fig. 7e). Consistent with the decreased infiltration in shTNFAIP8 AML mice, we also observed longer survival (Fig. 7g). Additionally, immunohistochemistry studies showed a partial lower expression of phosphorylated ERK in shTNFAIP8 AML mice, which is consistent with decreased activation of Rac1-ERK signaling caused by TNFAIP8 inhibition in vitro (Fig. 7f).

**Fig. 8:**
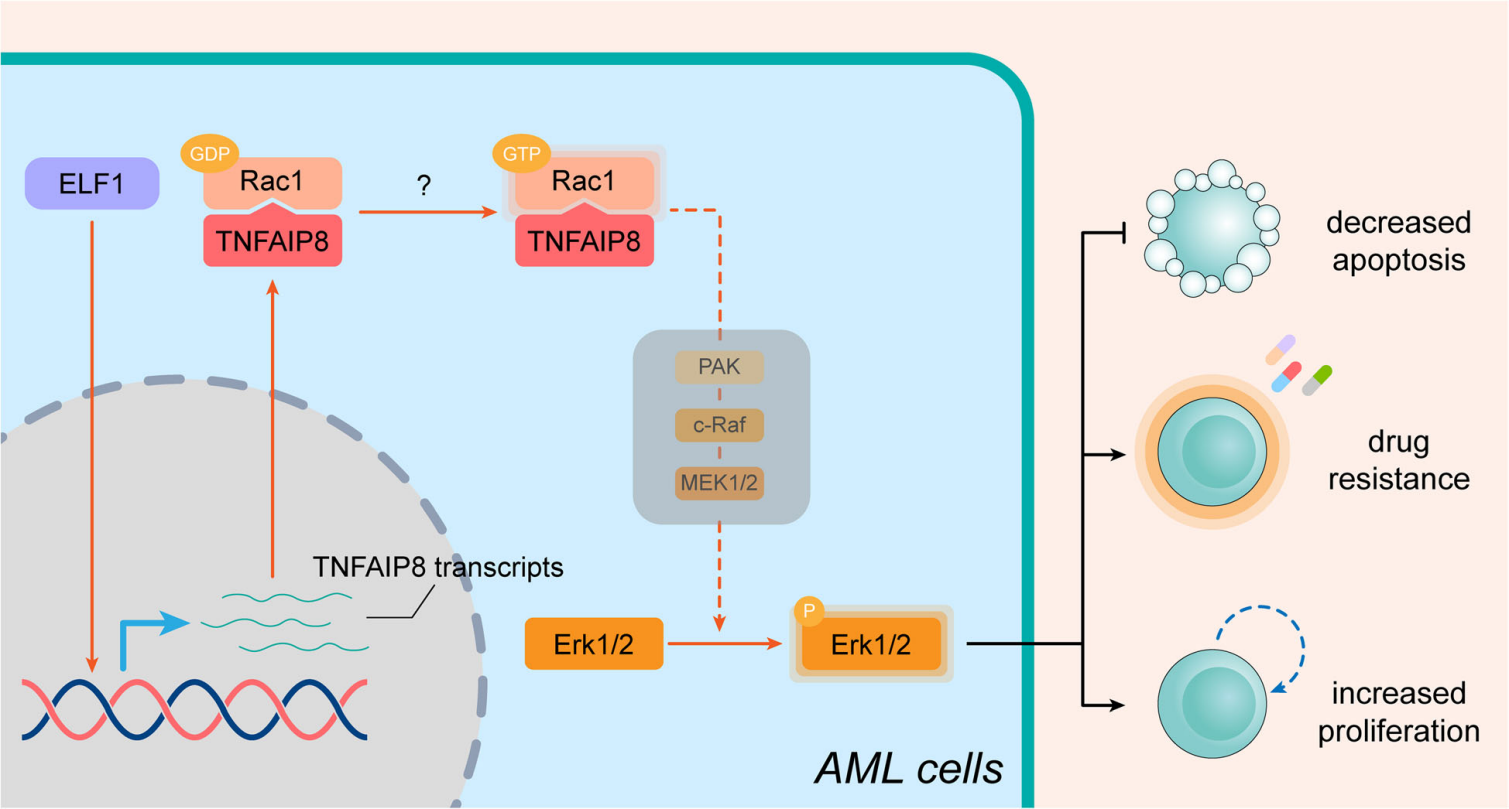
Schematic summary of this study. TNFAIP8 interacts with Rac1 and activates Rac1, an upstream factor of MAPK signaling pathway, making GDP-Rac1 transformed into GTP-Rac1, and upregulates ERK pathway ultimately. Activation of ERK pathway mediates AML cell proliferation, cell apoptosis blockage and chemoresistance, leading to leukemia progression ultimately

To sum up, we characterized the role for TNFAIP8 in AML chemoresistance and investigated its underlying molecular basis. We showed that TNFAIP8 suppresses apoptosis and promotes chemoresistance in AML by interacting with Rac1 to activate the ERK pathway (Fig. 8). Overall, these findings indicate that TNFAIP8 inhibition would be a promising therapeutic strategy for AML.

## Discussion

Evasion from apoptosis has been appreciated as one of the intrinsic mechanisms driving resistance to both traditional antineoplastic drugs and certain targeted therapies in malignant cells [46]. Previous experimental and clinical studies have focused on BCL-2 family members in AML [47]. However, the therapeutic values of narrowly targeting BCL-2 family in clinical settings have been compromised by occurrence of new resistance, partially due to functional compensation from non-targeted anti-apoptotic molecules [12–16]. It is of vital importance to uncover novel candidates. In the current study, we characterized the role for TNFAIP8, a new anti-apoptotic molecule, in AML chemoresistance and investigated its underlying molecular basis. We showed that TNFAIP8 suppresses apoptosis and promotes chemoresistance in AML by interacting with Rac1 to activate the ERK pathway. We also demonstrated that TNFAIP8 inhibition is effective against AML both in vitro and in vivo.

We found TNFAIP8 expression is higher in resistant AML cell lines than sensitive AML cell lines. In addition, TNFAIP8 expression was increased in relapsed/refractory AML patients compared with newly-diagnosed AML patients, implicating a possible relationship between TNFAIP8 expression and chemotherapeutic response. Future investigation on the clinical prognostic values of TNFAIP8 is warranted. Interestingly, we then identified a previously undescribed role for ELF1 in transcriptional regulation of TNFAIP8. ELF1 has been implicated in transcription regulation of several tumor-promoting genes including *Tie2*, *MEIS1*, *CCL2*, LUCAT1 [48–51]. Besides, data from TCGA suggest an increased ELF1 expression and a positive correlation between ELF1 and TNFAIP8 in AML, further supporting the contribution of ELF1 to upregulated TNFAIP8 expression. Since the significance and role for ELF1 in AML remain elusive, future investigation is worthy of consideration, which may help in expanding our understanding of the dysregulated molecular networks in AML.

A previous study has demonstrated high TNFAIP8 expression in acute leukemia cell lines, whereas its exact roles in AML remain understudied [18]. Here, we revealed the positive impact of TNFAIP8 on AML chemoresistance and found that TNFAIP8 suppression increased chemosensitivity through promoting chemotherapy-induced apoptosis in vitro. In addition, we found that mice bearing AML cells with TNFAIP8 suppression demonstrated lower leukemia infiltration and improved survival, providing in vivo experimental evidence for its therapeutic values. Together, our data suggest that TNFAIP8 might serve as a new candidate and justify investigating dual inhibition of TNFAIP8 and other anti-apoptotic molecules for mitigating AML chemoresistance in future studies.

Understanding the functional mechanism of TNFAIP8 in AML would greatly facilitate development of targeted therapy. Although the role of TNFAIP8 in negative regulation of apoptosis in malignancies have been well documented, the mechanisms by which TNFAIP8 functions vary among different cancer types and cell contexts [17–25]. Our study interpreted a previously unknown link between TNFAIP8 and ERK. We found that, in AML, TNFAIP8 regulated apoptosis and proliferation accompanied by altered phosphorylation of ERK1/2 and inhibition of ERK activation partially abrogated the down-regulation on the apoptotic level by overexpressing TNFAIP8. ERK, acting as the main downstream effector in MAPK signaling pathway, plays an important role in cell proliferation and survival. Elevated ERK1/2 phosphorylation has been found in 83.3% of AML patients [36], and inhibition of ERK1/2 induces cell cycle arrest and apoptosis in leukemic blasts [34, 52]. Notably, ERK1/2 inhibition can increase cell sensitivity to chemotherapeutics in AML [52, 53]. Given the significance of ERK signaling pathway in AML, our research provides novel insights that TNFAIP8 may promote AML chemoresistance by activating ERK signaling pathway. Rac1, a small GTPase, has been identified as a critical upstream mediator of the ERK pathway [40–45]. We found that TNFAIP8 promoted ERK phosphorylation through modulating Rac1. Interestingly, Rac1 is overexpressed in primary AML cells, and has gained attention for its roles in AML initiation and chemoresistance [54–56]. Our results shed light on the physical interaction between TNFAIP8 and Rac1. However, the mechanism underlying activation of TNFAIP8 on Rac1 remains unclear. Guanine nucleotide exchange factors (GEFs) have been shown to mediate the exchange of GDP for GTP by associating with membrane-bound Rac1, thereby activating Rac1 [57]. Thus, there is good reason to hypothesize that TNFAIP8 serves as a platform for interaction between GEFs and Rac1. The possibility that TNFAIP8 binds other membrane proteins to facilitate the plasma membrane location of Rac1 also merits further exploration.

## Conclusions

This is the first evidence for a role for TNFAIP8 in apoptosis regulation and drug resistance in AML. We provided experimental evidence that TNFIAP8, by interaction with Rac1, activated the ERK signaling pathway. Both in vitro and in vivo, our study strongly indicates that targeting TNFAIP8 is a promising strategy for overcoming AML chemoresistance.

## Supplementary information

**Additional file 1: Table S1.** Transcription factors that bound around TNFAIP8 gene.

**Additional file 2: Figure S1.** Representative immunoblotting analysis of TNFAIP8 in patients with newly-diagnosed AML (*n* = 5) and healthy control (CTR, *n* = 3).

**Additional file 3: Figure S2.** The expression levels of ELF1 in leukemia cell lines and the effect of ELF1 knockdown on TNFAIP8 expression. **a** RT-qPCR and immunoblotting analysis of ELF1 expression in leukemia cell lines (repeated three times). * *P* < 0.05, ** *P* < 0.01 means the drug resistant cell line (K562/A02 or HL60/ADR) versus the sensitive cell line (K562 or HL60). **b** TNFAIP8 and ELF1 expression in K562/A02 and HL60/ADR cells transduced with ELF1 shRNA (shELF1) or negative control (shNC) by RT-qPCR. Data are mean ± SD values of three independent experiments. * *P* < 0.05; ** *P* < 0.01.

**Additional file 4: Figure S3.** Caspase 3 and caspase 8 activation analysis in AML cells. Activation of Caspase 3 and caspase 8 was measured by western blot in HL60 or K562 transduced with Flag-tagged TNFAIP8 or control vector, and HL60/ADR or K562/A02 transduced with TNFAIP8 shRNA or negative control. Cells were stimulated with doxorubicin (1 μg/mL) for 24 h.

**Additional file 5: Figure S4.** The effects of downregulation of TNFAIP8 in THP-1 and U937. **a** TNFAIP8 knockdown (shTNFAIP8) or nonsilencing scrambled control (shNC) THP1 and U937 cells were selected by puromycin followed by RT-qPCR and western blots with indicated antibodies. **b** Proliferation of cells (shTNFAIP8 or shNC) were assessed by CCK8 assays, and proliferation rates at 0, 12, 24, 48 and 72 h were calculated normalized to the absorbance at 0 h. **c** Cells (shTNFAIP8 or shNC) were treated with ADM (1 μM), Ara-C (3 μM) and IDA (0.08 μM) for 48 h to measure apoptosis by flow cytometry. Data are mean ± SD values of three independent experiments calculated by Mann-Whitney U test or unpaired Student t-test. * *P* < 0.05; ** *P* < 0.01; *** *P* < 0.001.

**Additional file 6: Figure S5.** The effects of upregulation of TNFAIP8 in THP-1 and U937. **a** THP-1 and U937 were transduced with Flag-tagged TNFAIP8 (TNFAIP8) or control lentivirus (Mock). Cells were sorted by puromycin followed by quantitative PCR and western blots with indicated antibodies. **b** Proliferation of cells (TNFAIP8 or Mock) was assessed by CCK8 assays. **c** Cells (TNFAIP8 or Mock) were treated with ADM (1 μM), Ara-C (3 μM) and IDA (0.08 μM) for 48 h to measure apoptosis by flow cytometry. Data are mean ± SD values of three independent experiments calculated by Mann-Whitney U test or unpaired Student t-test. * P < 0.05; ** P < 0.01; *** P < 0.001.

**Additional file 7: Figure S6.** The influence of ADM-treating time and concentration on ERK1/2 activation. **a** The influence of Doxorubicin (ADM)-treating time on ERK1/2 activation was measured by western blot in K562. **b** The influence of ADM-treating concentration on ERK1/2 activation in K562 transduced with Flag-tagged TNFAIP8 or control vector.

**Additional file 8: Figure S7.** RNA-Sequencing analysis in TNFAIP8-knockdown cells. **a** Heat map for RNA sequencing assays in TNFAIP8-knockdown cells (shTNFAIP8) and negative control cells (shNC). Colors from red to green indicate high to low relative expression. *N* = 3. **b** Gene Pathway Relation Network depicting connections among differentially expressed KEGG gene sets. Red and blue represent upregulated and downregulated genes, respectively. Dot size represents significance. **c** KEGG pathway analysis of differentially expressed genes. **d** Gene ontology enrichment analysis of differentially expressed genes.

## Data Availability

All data available within the article and supplementary files, or available from the author upon request. Gene Expression Omnibus: all newly generated RNA-Sequencing data were deposited under accession number GSE154253.

## Abbreviations

ADM: Doxorubicin
AML: Acute myeloid leukemia
Ara-C: Cytarabine
BM: Bone marrow
ChIP: Chromatin immuno-precipitation
CR: Complete remission
DED: Death effector domain
ELF1: E74 like ETS transcription factor 1
ERK: Extracellular signal-regulated kinase
FAB: French-American-British classification
GEF: Guanine nucleotide exchange factors
HGB: Hemoglobin
HNSCC: Head and neck squamous cell carcinoma
IC_50_: Half maximal inhibitory concentration
IDA: Idarubicin
PAK: P21-activated kinase
PLT: Platelets
Rac1: Rac family small GTPase 1
RT-qPCR: Quantitative reverse transcription PCR
SD: Standard deviation
TNFAIP8: Tumor necrosis factor ɑ-induced protein 8
TSS: Transcription start site
WBC: White blood cells
