# Supplementary material for: TNFAIP8 promotes AML chemoresistance by activating ERK signaling pathway through interaction with Rac1
Source: J Exp Clin Cancer Res. 2020 Aug 14;39:158. doi: 10.1186/s13046-020-01658-z (PMC7427779; doi:10.1186/s13046-020-01658-z)
Supplement: Supplementary file 1 — Additional file 1: Table S1. Transcription factors that bound around TNFAIP8 gene. [file 13046_2020_1658_MOESM1_ESM.pdf]

Table S1 Transcription factors that bound around TNFAIP8 gene

Transcription factor	Official Full Name	Number of samples found (upstream)	Number of samples found (downstream)	Number of binding sites contain motif (upstream)	Number of binding sites contain motif (downstream)
ARNT	aryl hydrocarbon receptor nuclear translocator	0	1	0	1
ATF2	activating transcription factor 2	1	0	1	0
BARHL1	BarH-like homeobox 1	0	1	0	1
BCLAF1	BCL2-associated transcription factor 1	0	1	0	1
CEBPA	CCAAT/enhancer binding protein (C/EBP), alpha	0	2	0	2
CEBPD	CCAAT/enhancer binding protein (C/EBP), delta	0	1	0	1
CREB1	cAMP responsive element binding protein 1	4	0	4	0
CTCF	CCCTC-binding factor (zinc finger protein)	5	0	5	0
E2F1	E2F transcription factor 1	0	2	0	2
E2F4	E2F transcription factor 4, p107/p130-binding	0	2	0	2
E2F6	E2F transcription factor 6	0	2	0	2
EBF1	early B-cell factor 1	0	1	0	1
EGR1	early growth response 1	3	0	3	0
ELF1	E74-like factor 1 (ets domain transcription factor)	6	1	7	1
ELK3	ELK3, ETS-domain protein (SRF accessory protein 2)	1	0	1	0
ERG	v-ets avian erythroblastosis virus E26 oncogene homolog	1	1	1	1
ETS1	v-ets avian erythroblastosis virus E26 oncogene homolog 1	1	2	1	2
FOXA1	forkhead box A1	0	1	0	1
FOXD2	forkhead box D2	0	1	0	1
FOXP2	forkhead box P2	0	1	0	1
GATA1	GATA binding protein 1 (globin transcription factor 1)	0	1	0	1
GATA3	GATA binding protein 3	0	2	0	2
GATA4	GATA binding protein 4	0	1	0	1
GATA6	GATA binding protein 6	0	2	0	2
GMEB2	glucocorticoid modulatory element binding protein 2	1	0	1	0
HOXC6	homeobox C6	0	1	0	1
IRF1	interferon regulatory factor 1	0	1	0	1
KDM5A	lysine (K)-specific demethylase 5A	1	0	1	0
KDM5B	lysine (K)-specific demethylase 5B	0	1	0	2

KLF4	Kruppel-like factor 4 (gut)	1	0	1	0
KLF5	Kruppel-like factor 5 (intestinal)	1	1	1	1
LEF1	lymphoid enhancer-binding factor 1	0	1	0	1
MTA3	metastasis associated 1 family member 3	1	0	1	0
MYB	v-myb avian myeloblastosis viral oncogene homolog	1	0	1	0
MYBL2	v-myb avian myeloblastosis viral oncogene homolog-like 2	0	1	0	1
MYC	v-myc avian myelocytomatosis viral oncogene homolog	1	1	1	1
NFYA	nuclear transcription factor Y subunit alpha	1	0	1	0
NFYB	nuclear transcription factor Y subunit beta	2	0	2	0
PAX5	paired box 5	1	1	1	1
RARG	retinoic acid receptor, gamma	1	0	1	0
RCOR1	REST corepressor 1	0	1	0	1
RELA	v-rel avian reticuloendotheliosis viral oncogene homolog A	0	2	0	2
RFX1	regulatory factor X1	1	0	1	0
SNAI2	snail family zinc finger 2	0	4	0	4
SP1	Sp1 transcription factor	1	0	1	0
SP3	Sp3 transcription factor	0	1	0	1
SP4	Sp4 transcription factor	1	0	1	0
STAT4	signal transducer and activator of transcription 4	1	0	1	0
STAT5A	signal transducer and activator of transcription 5A	1	2	1	2
TAL1	T-cell acute lymphocytic leukemia 1	0	4	0	4
TBP	TATA-box binding protein	0	1	0	1
TFDP2	transcription factor Dp-2 (E2F dimerization partner 2)	0	1	0	1
TP63	tumor protein p63	0	5	0	5
UBTF	upstream binding transcription factor, RNA polymerase I	0	1	0	1
YY1	YY1 transcription factor	6	1	6	1
ZFP42	ZFP42 zinc finger protein	0	1	0	1

ChIPBase v2.0 project, hg38, the statistics of transcription factors that bound around TNFAIP8(ENSG00000145779.7) gene
The number column below is corresponding to the motif status
