# Supplementary figures and images for: TNFAIP8 promotes AML chemoresistance by activating ERK signaling pathway through interaction with Rac1
Source: J Exp Clin Cancer Res. 2020 Aug 14;39:158. doi: 10.1186/s13046-020-01658-z (PMC7427779; doi:10.1186/s13046-020-01658-z)

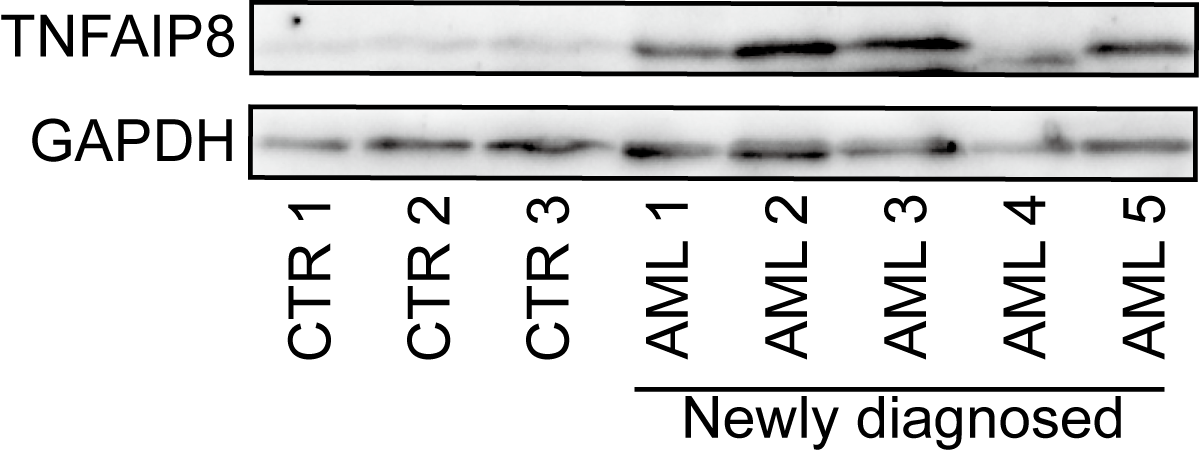

Supplement: Supplementary file 2 — Additional file 2: Figure S1. Representative immunoblotting analysis of TNFAIP8 in patients with newly-diagnosed AML (n = 5) and healthy control (CTR, n = 3). [file 13046_2020_1658_MOESM2_ESM.tif]

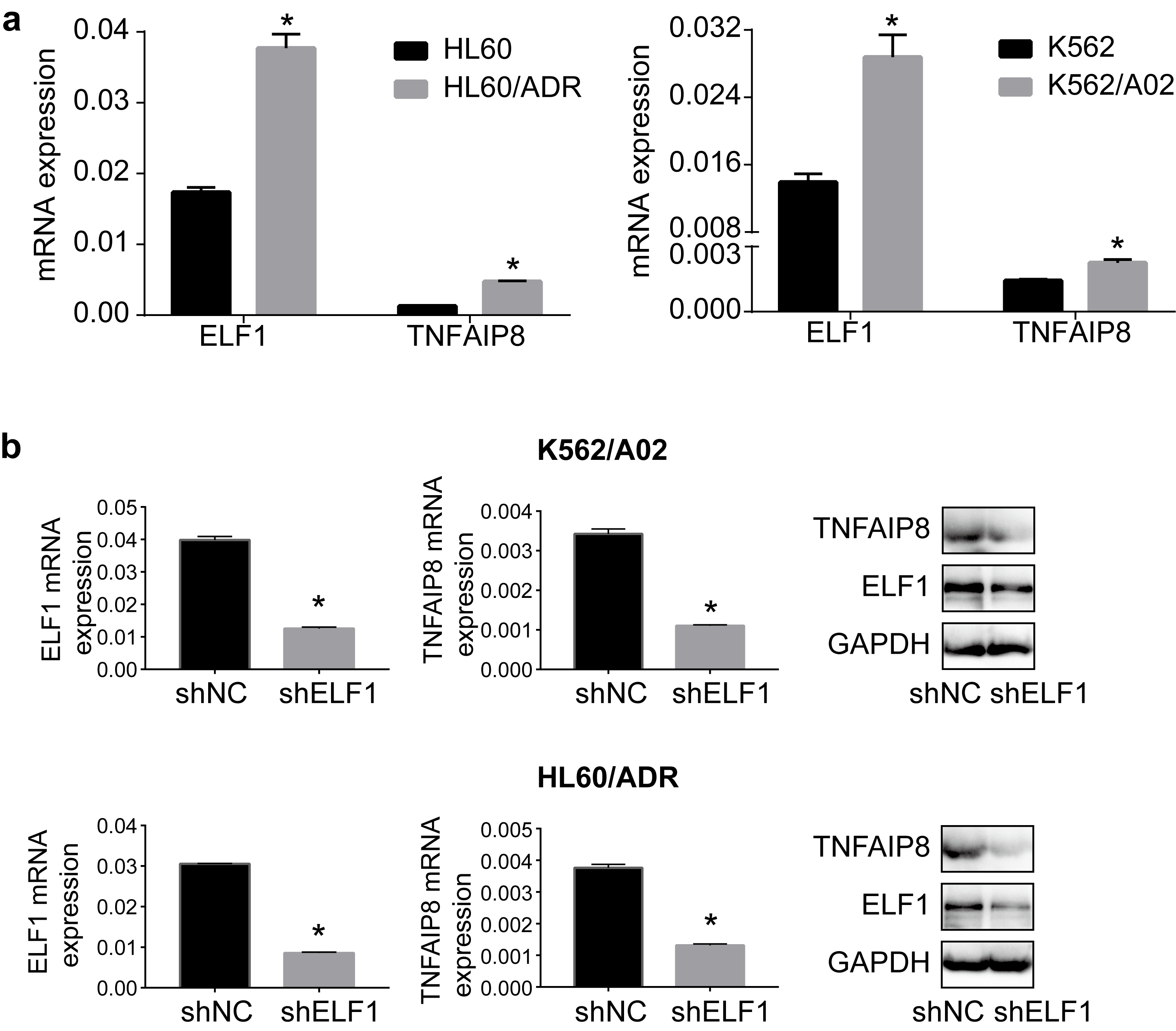

Supplement: Supplementary file 3 — Additional file 3: Figure S2. The expression levels of ELF1 in leukemia cell lines and the effect of ELF1 knockdown on TNFAIP8 expression. a RT-qPCR and immunoblotting analysis of ELF1 expression in leukemia cell lines (repeated three times). * P < 0.05, ** P < 0.01 means the drug resistant cell line (K562/A02 or HL60/ADR) versus the sensitive cell line (K562 or HL60). b TNFAIP8 and ELF1 expression in K562/A02 and HL60/ADR cells transduced with ELF1 shRNA (shELF1) or negative control (shNC) by RT-qPCR. Data are mean ± SD values of three independent experiments. * P < 0.05; ** P < 0.01. [file 13046_2020_1658_MOESM3_ESM.tif]

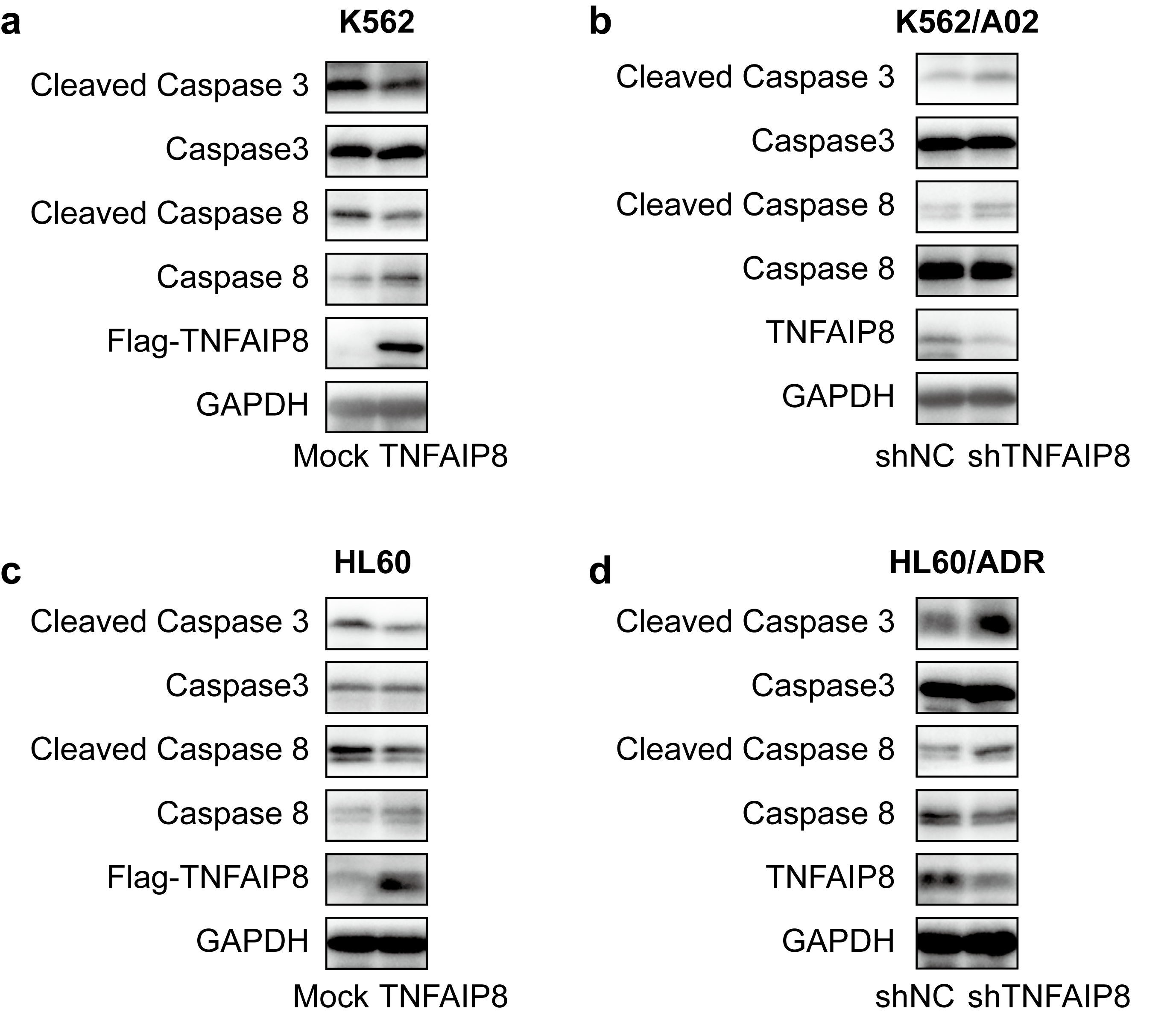

Supplement: Supplementary file 4 — Additional file 4: Figure S3. Caspase 3 and caspase 8 activation analysis in AML cells. Activation of Caspase 3 and caspase 8 was measured by western blot in HL60 or K562 transduced with Flag-tagged TNFAIP8 or control vector, and HL60/ADR or K562/A02 transduced with TNFAIP8 shRNA or negative control. Cells were stimulated with doxorubicin (1 μg/mL) for 24 h. [file 13046_2020_1658_MOESM4_ESM.tif]

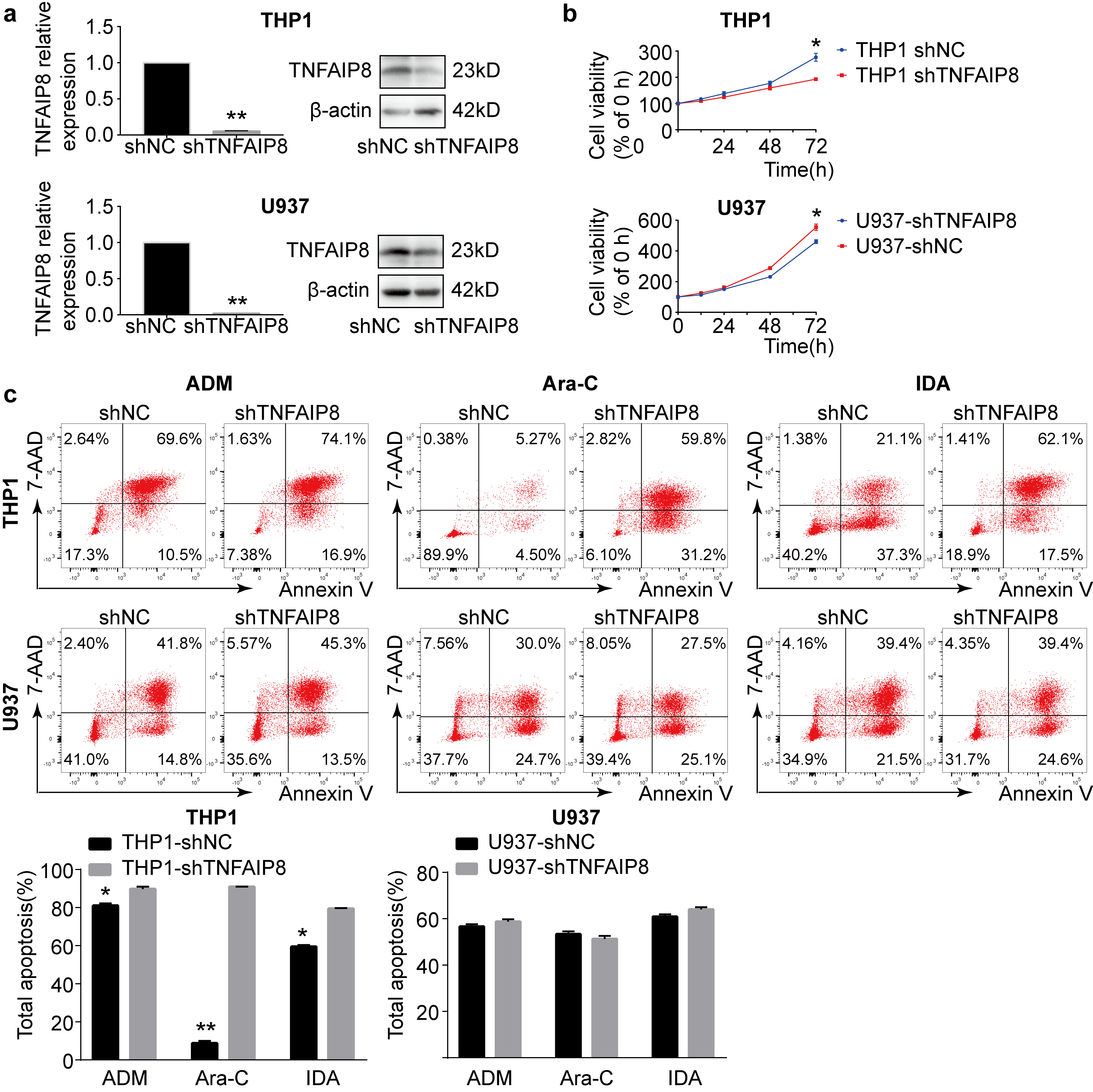

Supplement: Supplementary file 5 — Additional file 5: Figure S4. The effects of downregulation of TNFAIP8 in THP-1 and U937. a TNFAIP8 knockdown (shTNFAIP8) or nonsilencing scrambled control (shNC) THP1 and U937 cells were selected by puromycin followed by RT-qPCR and western blots with indicated antibodies. b Proliferation of cells (shTNFAIP8 or shNC) were assessed by CCK8 assays, and proliferation rates at 0, 12, 24, 48 and 72 h were calculated normalized to the absorbance at 0 h. c Cells (shTNFAIP8 or shNC) were treated with ADM (1 μM), Ara-C (3 μM) and IDA (0.08 μM) for 48 h to measure apoptosis by flow cytometry. Data are mean ± SD values of three independent experiments calculated by Mann-Whitney U test or unpaired Student t-test. * P < 0.05; ** P < 0.01; *** P < 0.001. [file 13046_2020_1658_MOESM5_ESM.tif]

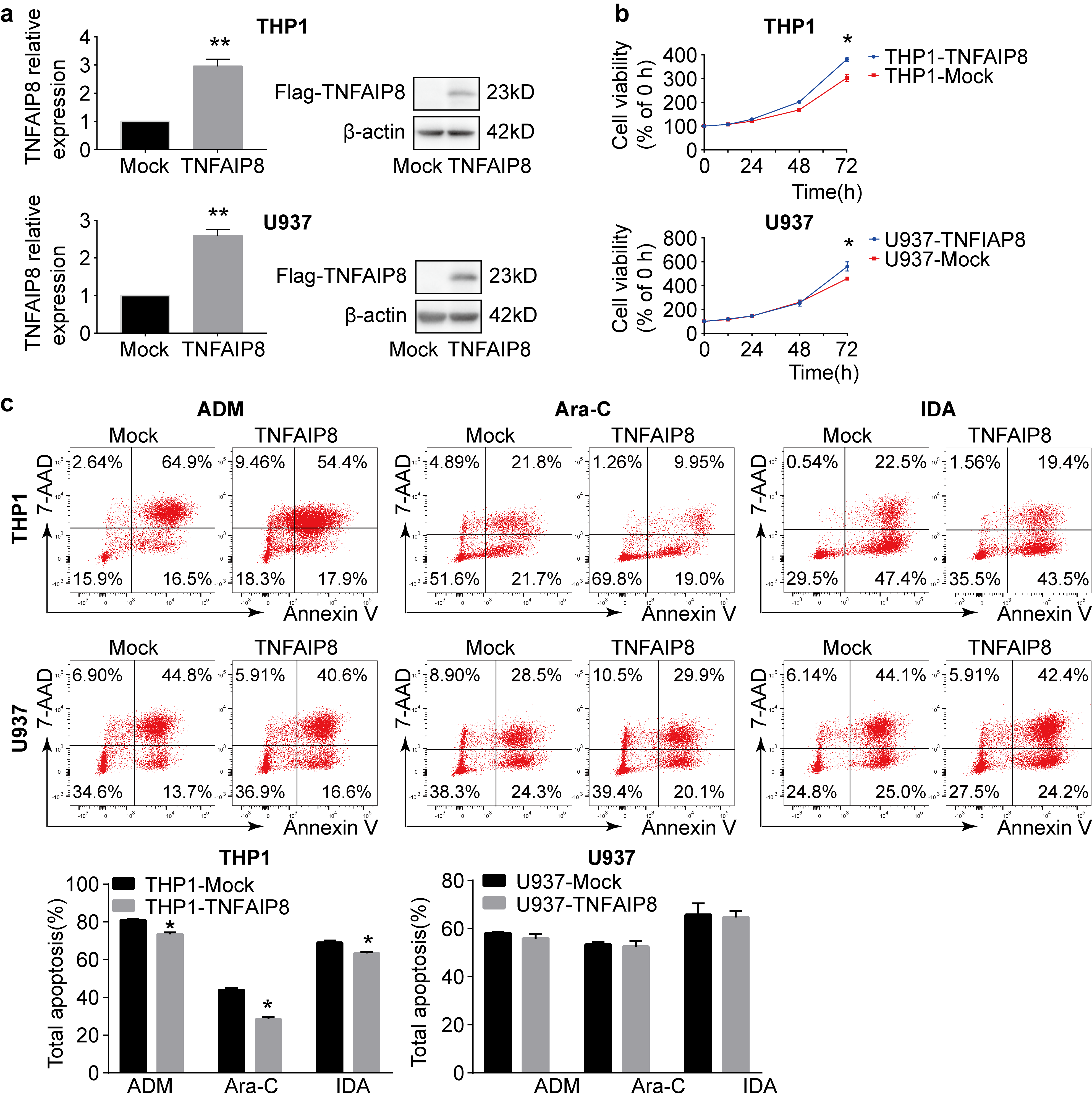

Supplement: Supplementary file 6 — Additional file 6: Figure S5. The effects of upregulation of TNFAIP8 in THP-1 and U937. a THP-1 and U937 were transduced with Flag-tagged TNFAIP8 (TNFAIP8) or control lentivirus (Mock). Cells were sorted by puromycin followed by quantitative PCR and western blots with indicated antibodies. b Proliferation of cells (TNFAIP8 or Mock) was assessed by CCK8 assays. c Cells (TNFAIP8 or Mock) were treated with ADM (1 μM), Ara-C (3 μM) and IDA (0.08 μM) for 48 h to measure apoptosis by flow cytometry. Data are mean ± SD values of three independent experiments calculated by Mann-Whitney U test or unpaired Student t-test. * P < 0.05; ** P < 0.01; *** P < 0.001. [file 13046_2020_1658_MOESM6_ESM.tif]

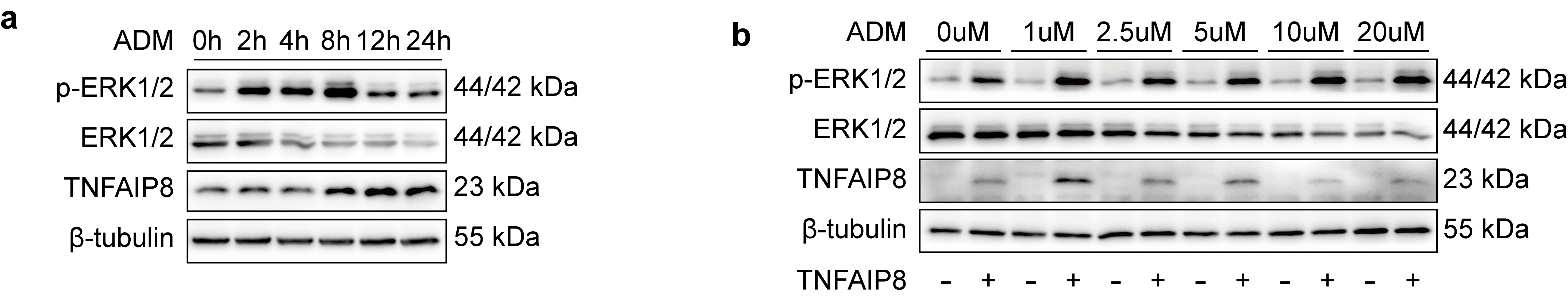

Supplement: Supplementary file 7 — Additional file 7: Figure S6. The influence of ADM-treating time and concentration on ERK1/2 activation. a The influence of Doxorubicin (ADM)-treating time on ERK1/2 activation was measured by western blot in K562. b The influence of ADM-treating concentration on ERK1/2 activation in K562 transduced with Flag-tagged TNFAIP8 or control vector. [file 13046_2020_1658_MOESM7_ESM.tif]

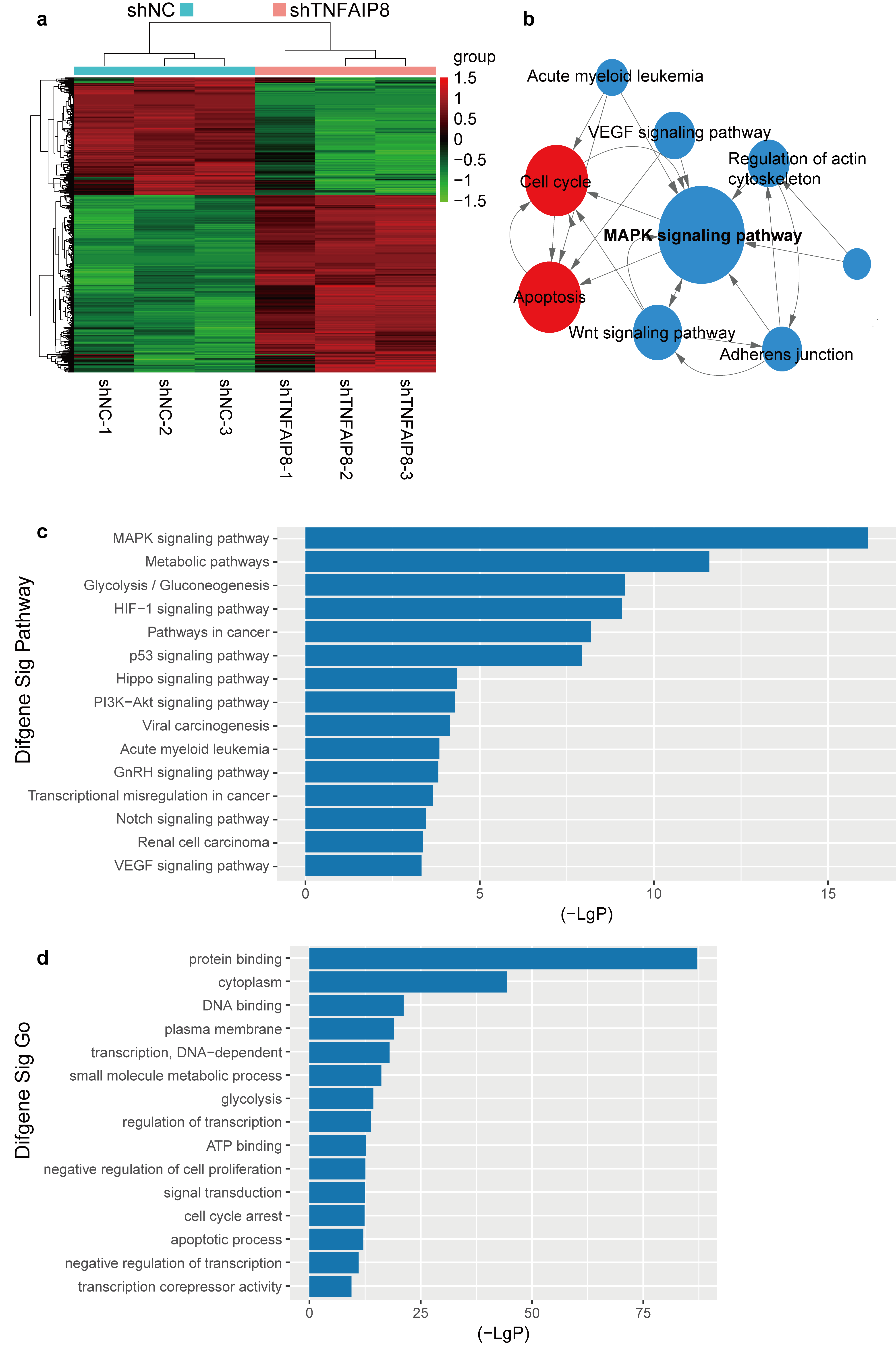

Supplement: Supplementary file 8 — Additional file 8: Figure S7. RNA-Sequencing analysis in TNFAIP8-knockdown cells. a Heat map for RNA sequencing assays in TNFAIP8-knockdown cells (shTNFAIP8) and negative control cells (shNC). Colors from red to green indicate high to low relative expression. N = 3. b Gene Pathway Relation Network depicting connections among differentially expressed KEGG gene sets. Red and blue represent upregulated and downregulated genes, respectively. Dot size represents significance. c KEGG pathway analysis of differentially expressed genes. d Gene ontology enrichment analysis of differentially expressed genes. [file 13046_2020_1658_MOESM8_ESM.tif]
